# The analytical landscape of static and temporal dynamics in transcriptome data

**DOI:** 10.3389/fgene.2014.00035

**Published:** 2014-02-20

**Authors:** Sunghee Oh, Seongho Song, Nupur Dasgupta, Gregory Grabowski

**Affiliations:** ^1^Division of Human Genetics, Department of Pediatrics, Cincinnati Children’s Hospital Medical CenterCincinnati, OH, USA; ^2^Department of Mathematical Sciences, McMicken College of Arts and Sciences, University of CincinnatiCincinnati, OH, USA

**Keywords:** RNA-seq, initial digital technology, microarray, gene expression, differential expression, static and temporal dynamics

## Abstract

Interpreting gene expression profiles often involves statistical analysis of large numbers of differentially expressed genes, isoforms, and alternative splicing events at either static or dynamic spectrums. Reduced sequencing costs have made feasible dense time-series analysis of gene expression using RNA-seq; however, statistical methods in the context of temporal RNA-seq data are poorly developed. Here we will review current methods for identifying temporal changes in gene expression using RNA-seq, which are limited to static pairwise comparisons of time points and which fail to account for temporal dependencies in gene expression patterns. We also review recently developed very few number of temporal dynamic RNA-seq specific methods. Application and development of RNA-specific temporal dynamic methods have been continuously under the development, yet, it is still in infancy. We fully cover microarray specific temporal methods and transcriptome studies in initial digital technology (e.g., SAGE) between traditional microarray and new RNA-seq.

## MOVEMENT IN DIFFERENTIAL ANALYSIS WITH New-ERA RNA-seq

The primary goal of whole-transcriptome analysis is to identify, characterize, and catalog all the transcripts expressed within a specific cell or multiple tissues, either at a static given stage or across dynamic time-varying stages. As microarray, there are major two distinct types of experimental design for identification of differentially expressed genes in RNA-seq (Leek et al., 2006). *The first* type of data analysis is to compare simple pairwise group comparisons from a static sampling experiment where samples are collected from distinct biological groups without respect to time. In this setting, there are typically two-group comparison and multi-group comparison (more than two). *The second type* of experimental design in gene expression profiling is a temporal experiment with or without replicates, where samples are collected over a time window to characterize temporal dynamic spectrum and underlying developmental or progressive biological mechanisms. In this category, large-scale longitudinal data with repeated measurements in gene expression profiles are also additionally included. Temporal dynamic studies in disease progression and age- related psychiatry data (e.g., stimulated aggressive spectrum disorders and neurodegenerative disorders). Otto et al. (2010), Prensner et al. (2011), Lv et al. (2013), Palmblad et al. (2013) and Sharron Lin et al. (2013) have been substantially studied more recently following by static expression profile study as microarray. Hence, it is very timely crucial to comprehensively review existing methods with pros and cons and guide future direction on analytical strategy and application of methods in static and temporal dynamics. Time-series experiment is largely composed of three different settings, (I) a single-series time course to study a developmental transient pattern (Pauli et al., 2012), (II) a multi-series or factorial time course that interrogates multiple biological reactions to specific external stimuli at each time point (Jager et al., 2011; Sivriver et al., 2011), and (III) a periodical time course in cell-cycle or circadian rhythmic data (Bar-Joseph et al., 2012; Cheng et al., 2013; Lokody, 2014).

In recent studies, some statistical methodologies and corresponding R packages at a static time point in RNA-seq to address *the first type* of data have been proposed, such as Fisher’s exact test (Fisher, 1941), Audic–Claverie statististics (Tino, 2009), DEGSeq with normal approximation from binomial distribution (Wang et al., 2010), edgeR (Robinson et al., 2010), and DESeq (Anders and Huber, 2010) for simple pairwise comparison between one sample without replicates vs. another sample, or one group vs. another group when there are replicates for each group.

Recently, DESeq and edgeR have further incorporated multi-group comparisons (Anders and Huber, 2010; Oshlack et al., 2010). DESeq is roughly the same principle as edgeR; the main difference is in the estimation of dispersion for variation of replicates and in incorporating their own normalization procedures. More recently, edgeR and DESeq have provided a generalized linear model (GLM) approach incorporated in their R packages as a pooling method of samples. A pooling method is to take all samples and factors (e.g., tissue and replicate factor) in the model simultaneously and to test a hypothesis of difference on a major biological interest (tissue difference) by controlling secondary nuisance factors (variability of replicates). They enable to test RNA-seq gene expression profiles with a more complicated experimental design having multiple nuisance factors to be controlled in the model as well as major biological interest (or more than one) to be tested in the differential expression (DEX) analysis, for instance, the difference of age, sex, species, replicates, or other experimental factors.

To overcome the small sample size issue in RNA-seq experimental design that is currently common practice, by utilizing prior information across genes, such as Bayesian approaches, baySeq (Hardcastle and Kelly, 2010), Bayesian model-based DEX (BM-DE; Lee et al., 2011), shrinkage method (Smyth, 2004; Li and Tibshirani, 2011; Zhou et al., 2011; Wu et al., 2013), and more recently, a distribution-free approach and non-parametric approach (Li et al., 2012; Li and Tibshirani, 2013) have been introduced into the differential analysis of an RNA-seq experiment. The main goal of baySeq with gamma-Poisson and empirical negative binomial distribution is to compare two or multiple groups. Their approaches are based on an empirical Bayesian approach to estimate posterior probabilities of patterns of differential or equal expression for a given gene expression profile and prior knowledge. Poisson-gamma model approach in baySeq is to assume that the read count data (or normalized expression data) are poisson distributed and prior parameters follow gamma distribution. Another possible model with negative binomially (over-dispersed poisson) distributed data has been also proposed in the paper since Poisson model fails to account for the extra variability of biological replicate on samples. The BM-DE method is another Bayesian approach to apply with yeast data of strain BY4741 containing two different groups to be compared, in a rich growth medium and a poor growth medium. This proposed method is on the basis of individual nucleotide position level read counts other than unified gene level read counts by accounting for extreme position-specific outliers of expression on samples. Namely, the method quantified expression levels of a genomic position within a gene by considering over-dispersion on different positions in a gene using hierarchical modeling with beta distribution to allow biological replicates and dirichlet and gamma prior. They evaluated and demonstrated the proposed position level method shows better performance when compared existing methods, DEGSeq proposed by Wang et al. (2010) and analysis of sequence counts proposed by Wu et al. (2010).

In RNA-seq, the most recent distribution-free method, a rank-based non-parametric method proposed by Li and Tibshirani (2013) is to identify a set of differentially expressed transcripts/genes in simple pairwise two-comparison or multiple-group comparison based on a particular given static stage. Interestingly, their methods also address the identification problems of altered gene expression in survival data using score statistics and the multiple resampling procedures as a semi-parametric method other than Breslow’s method and Cox-proportional model. Current static methods have been also compared in comparative studies to evaluate the performance of proposed methods (Bullard et al., 2010; Kadota et al., 2012; Kvam et al., 2012). However, no consensus was achieved and no unanimously conclusive best method exists, and the work can be expanded to cover generalizability for various types of RNA-seq data. Thus there is a need for a better methodology to preserve elegant discrete distributions for counts other than converting variance-stabilizing transformation from counts in sequencing experiments to continuous measurements in array-based experiments – although there is no intrinsic advantage to having discrete numbers rather than continuous ones in gene expression profiling (Douglas and Wood, 2011; Meyer et al., 2012).

Rather than preserving an elegant digital measure, i.e., the discreteness of the expression levels of mapped read counts onto a reference genome, by borrowing statistical methods from microarray through a variance-stabilizing transformation, a linear models for microarray (LIMMA) procedure has been proposed as another pooling method. However, when the read counts of expression are relatively large, methods based on the transformation and consequence of standard normal Gaussian approaches fit very well. When expression levels have small read counts, such approximated asymptotic approaches from the transformation of discrete counts into continuous variable are less accurate. The main drawback of pooling methods, the GLM, or LIMMA approach for time-series RNA-seq data is that even if the labels of a sample from one time point to another time point are reordered, the results would be identical based on *F*-statistics. Since the sample size in the RNA-seq experiment is much smaller than in microarray, identification of DEX through GLM-based approaches suffers from power issue of detection.

To consider *the second type* of experimental in RNA-seq, these dynamic processes are more likely to be related to underlying mechanisms of disease progression and developmental process. Nevertheless, application and development of method on RNA-seq data have mostly focused on static two-group or multi-group comparisons, whereas, more complicated experimental design settings such as temporal dynamics have not been addressed. Due to the lack of methods to precisely analyze temporal dynamics, as alternative and intuitive solutions, existing (I) pairwise methods (II) pooling analysis of sample to run all samples simultaneously in the model, and (III) clustering analysis to group co-expressed similar patterns. Mainly, there are three different types of time course experiment: (1) a single-series of time course to explore single temporal transient pattern, (2) a multi-series of time course to simultaneously explore differences on expression levels among biological conditions in vertical line and expression patterns over time within a condition to define horizontal temporal trajectory, as a state-of-art visualization, consecutive patterns of vertical differences by given conditions can be shown in temporal fashion whether there is at least one pair to be different on the multiple conditions over a series of time points. This multi-series of time course expression profile has been widely performed in stimulus-response time-series experiment to identify altered expression of multiple responses from different conditions of stimuli. (3) Another type of time course is periodicity including cell-cyclic and circadian rhythmic patterns. The periodical time course data can be also incorporated with multiple factors at a time. In microarray factorial cell-cycle time-series data, Rosa et al. (2012) demonstrated identification of treatment, clock, and noise frequency. Future work on these issues will prompt the development of common guidelines to identify and characterize changes of altered expression over time in RNA-seq, similar to the guidelines established for time-series microarrays. In this section, we describe the fundamental advantages and disadvantages of existing methods by focusing on comprehensively illustrating more recent efforts on static DEX methods and clustering techniques in RNA-seq. Additionally, various dynamic methods in analog array-based and initial digital technology [e.g., SAGE (serial analysis of gene expression)] as RNA-seq has been replacing the older technologies rapidly.

## INVESTIGATING RNA-seq TIME-SERIES DATA USING EXISTING STATIC DEX METHODS

Most recently published studies of time course experiments in RNA-seq are inconclusive in terms of statistical viewpoints to infer temporal patterns (Anders and Huber, 2010; Jiao and Meyerowitz, 2010; Otto et al., 2010; Parikh et al., 2010; Graveley et al., 2011; Jager et al., 2011; Koike et al., 2012; Pauli et al., 2012). As prompt and alternative, temporally differential expression (TDE) has been identified by separately doing pairwise comparison between two neighboring time points and merging all possible pairwise comparisons into either union set (global set) or intersection set (stringent set) in their studies. Yet, such approaches do not explicitly take inherent time dependency structure into account, albeit it is certain that successive expression profiles are quite correlated during progressive dynamic regulation. Experimental and molecular genome-wide studies related with dynamic process have been conducted to explore developmental progression mechanisms on the basis of gene expression profile over time. Time-series experiment facilitates various different experimental settings as described in the previous section. More specifically, complicated factorial time course data with more conditions and factors have been performed to investigate altered changes on expression after external stimulation over time. And it is referred to as multi-series of time course and the conditions can be incorporated into either general time course or periodical data, namely, a stimulus-response time course with one or several experimental conditions at a time (general multi-series of time course) and periodical time course with multiple conditions (cell-cyclic multi-series of time course). In general, when analyzing time-series expression profile data, inference of estimates on DEX is not practically feasible or frequently obtaining very low power owing to few time points and replicates. Due to the limitation of application to time-series RNA-seq, it is good practice to perform gene clustering that might be more efficient in identifying groups of co-expressed temporal genes, as DEX methods are not competitive due to the power issue. However, it is also worth noting that clustering techniques are not designed to assess and order genes on a statistically significant timing difference between conditions for such complicated time course data. In addition, as intrinsic issues on clustering methods, the choice of the proper number of clusters and the visualization of a large number of genes can be problematic in running data and interpreting clustering results.

With the popularity of such rich systematic resources and reduced costs in profiling gene expression, it is clear that the complicated accompanying experiments will have multiple factors and parameters, e.g., the transcriptomic experiments with time window lie in the coming years. To address this type of data, the formal statistical tests used in temporal analysis will be more advantageous for understanding the causes and consequences of various human diseases observed over time in clinical applications or developmental processes. This manuscript comprehensively reviews statistical methodologies by focusing on a variety of dynamic time-series expression profiles in RNA-seq and outlines important questions in the field, speculating on how these statistical methods can analyze and interpret time-series expression profiles in aspects of human disease progression and on the accompanying evolutionary implications (Smith et al., 2009; Trapnell et al., 2010; Bar-Joseph et al., 2012; Oh et al., 2013). These issues are complex and poorly understood in biomedical research. Static and dynamic approaches differ markedly in required assumptions and in determining temporally differentially expressed genes.

It is now well known that a developmental transcriptome is highly dynamic and that the previous gene expression profile can affect the subsequent one, which in turn can influence the pattern of DEX. Currently, one of most popular computational bioinformatics tools for researchers in this field for identifying gene- and transcript-level expression is Cufflinks (Trapnell et al., 2010), where DEX analyses for simple pairwise two-group comparisons are allowed to perform by running Cuffdiff; this is not currently applicable, however, for comparisons with multi-groups and factors and by running Cuffcomp. In its current version, there is a time-series option for cell-cycle data, but it can only do simple pairwise comparisons from one time point to the next. Of course, based on the DEX union gene set from all possible simple comparisons, we can roughly identify the temporal expression of a given time-series; however, the union of marginal temporal expression does not guarantee overall temporal dynamics in time-series experiments. Thus dynamic-specific methods in the identification of temporal patterns across all time points have not been shown thus far to identify the overall dynamics and the developmental temporal expression over a time-series when compared to approaches of simple DEX genes between two-group comparisons. A fascinating distribution-free method based on the Wilcoxon rank test and the resampling method proposed by Li and Tibshirani (2013) can be applied for various types of experimental design. It is very robust when there are outlier samples of data compared to the previously mentioned recent differential parametric methods. However, it is also a static-based strategy, where real datasets were collected: Marioni data with five biological replicates, ‘t Hoen data (‘t Hoen et al., 2008) with four biological replicates, Witten data (Witten et al., 2010) with 29 biological replicates, and simulation studies with a moderate sample size (*n* = 12) and a small sample size (*n* = 5). Unfortunately, most current RNA-seq experiments have at most three replicates in common use. Although the cost of sequencing has dropped substantially, and experimental designs with appropriate replicates, depending on the hypotheses of the studies, have been suggested in terms of power and reliable outcomes, there are still very few biological replicates available in RNA-seq experiments, either static or dynamic.

In summary, the initial statistical techniques as indirect temporal dynamic methods provided a first glimpse into the identification of the DEX of gene regulation in RNA-seq in terms of simple pairwise comparison and multi-group/factor comparison. However, as interest grows in a variety of species and cell types, and scientific questions are asked about developmental stages, single cell-cyclic, and circadian rhythmic regulation. RNA transcriptome studies to identify TDE are involved with single-series, multi-series, and periodicity. Various time course experimental designs are gradually revealing relevant biological mechanisms and functional pathways in temporal dynamic process as in conventional microarray. Therefore, the development and application of statistical methodologies to uncover and capture the TDE across dynamic time points is timely very critical in RNA-seq. This is a comprehensive review article of static and dynamic algorithms by including oscillations in cell-cycle as well as computational modeling in traditional technologies. The field is advancing so rapidly that a brief review cannot include all of the work done in the past 5 years. It is a sampling of a few highlights of statistical methodologies in differential analysis, from conventional hybridization-based array experiments that provide a continuous fluorescent-intensity gene-expression profile to attractive new RNA-seq technology with count-based measurements from tag sequence in both static and dynamic time course experimental design settings.

## DIFFERENTIAL ANALYSIS WITH TIME COURSE METHODS IN MICROARRAY

Time course cDNA microarray experiments have been widely used to study temporal profiles of cell dynamics from a genomic perspective and to discover the associated gene regulatory relationship. The output gene expression matrix in a hybridization-based microarray is basically a large-scale dataset filled with numbers related to the signal fluorescent intensity between each gene and a sample on the chip. These raw data are pre-processed in a lower analysis, such as background detection, normalization, probe set summarization, and filtering out genes or outlier samples with specific criteria. For instance, the coefficient of variation is used to filter out genes which are less than the user-set cut-off value, and the missing values between genes and samples are estimated by imputing methods. The potential outlier samples with bad quality control measures are discarded beforehand. Some exploratory approaches, such as box-plot, mean-difference plot, correlation-plot, and principal component analysis, can be performed as diagnostic measures of outliers in a visualization manner. After such exploratory analyses, genes and samples that are of good quality and more meaningful of targets for further exploration are kept for downstream analyses, including identification of DEX at a static or dynamic mechanism in cellular responses over time. Once a certain set of genes are identified to be of interest for further investigation, grouping of clusters of genes with similarly co-expressed regulation patterns and analysis of pathway and gene ontology can be performed to identify biologically meaningful information and biologically related and interacting gene regulatory networks, e.g., in disease with respect to a normal control sample at a static time point or across a time-series or developmental time stages or covering expressions that are periodically regulated across the cell-cycles.

When replicated time course microarray data are available, various statistical approaches and modifications are employed. This category of approaches, ANOVA (Ahdesmaki et al., 2007; Graveley et al., 2011; Swan et al., 2011), has been extended to work with longitudinal data, where the microarray measurements lie in multi-dimensional space with the coordinates to a time point to be a correlated structure. It is a sequence of data points resulting from measurements which are recorded at successive moments, either equally evenly spaced in time or at irregular time points. However, replication of time-series or longitudinal sampling is costly if the number of time points is comparatively large. For this reason, many published time course datasets in microarrays have suffered from a relatively small number of samples compared to variables of genes when developing powerful methods. In this case, intuitively clustering-based approaches are applied first; model-specific approaches in small sample sizes have been also applied for ranking temporal biomarker genes and examining such temporal expression patterns. Basically, clustering-based approaches select genes whose patterns are similar to each other to find groups with co-expressed patterns, an unsupervised approach, as we do not have prior knowledge of gene expression patterns. For this purpose in microarray, some groups (Chu et al., 1998; Eisen et al., 1998; Ahdesmaki et al., 2007) have proposed a hierarchical clustering method, and (Tamayo et al., 1999; Saban et al., 2001), and (Burton et al., 2002) presented self-organizing maps and model-based clustering methods. Bar-Joseph et al. (2003) and Ramoni et al. (2002) performed Bayesian model-based clustering. Schliep et al. (2003) suggested the HMM clustering method.

When characterizing temporal features in a time course data, there are some drawbacks to merely considering clustering methods, in that they make no explicit use of the replicate information and they use all the slides or means of the replicates. That is, the clustering technique does not reveal what genes are differentially expressed among different experimental conditions and temporal patterns of such DEX sets. Another limitation in gene clustering methods for time course data is that clustering does not provide such ranking for the individual genes based on the magnitude of change in expression levels over time, which scientific researchers frequently want to investigate. Large microarray data sets with 20K ~ 40K genes are very common; however, clustering methods are not suited to handle such large input data files and may not provide clear group patterns due to the inevitable noisy set contained in microarray experiments.

Thus in microarray analog-based experiment platforms, diverse strategies were developed and applied in the literature to address different aspects of biological questions in time course gene expression data. For many applications of new digital-based RNA-seq technology, which has been rapidly replacing array-based experiments in transcriptome gene expression profiling, however, we are not able to simply plug in methods used in microarray, as it quantifies discreteness of expression level and somehow different biases from experiments and normalization strategies to adjust artifacts. More sophisticated RNA-seq-specific algorithms and software tools are particularly important in analyzing various RNA-seq applications along with the wave of data produced by this fast-moving RNA-seq technology. Statistical methodologies currently lack solutions to analyze time course experiments as well as other types of outcomes. Significant efforts must be undertaken on the statistical and computational methodology front. Sophisticated, tailor-made data analysis approaches will likely play a key role in fully realizing the power to interpret whole stories of the time-series RNA-seq transcriptome in next-generation sequencing technologies.

### PERIODICITY IN MICROARRAY AND RNA-seq

Since the pioneering work in methods of identification of periodically expressed genes as a function of the cell-cycle or circadian rhythm in microarray, various organisms at a genome-wide level have been studied in yeasts, plants, and mammals (Eisen et al., 1998; Wichert et al., 2004; Ptitsyn et al., 2007; Michael et al., 2008; Zhao et al., 2009; Geyfman et al., 2012; Oishi, 2012; Sporl et al., 2012). Using cell-cycle or circadian rhythmic data from microarray has long been a critical modeling approach in studying periodical transcriptional regulation statistically. With the new RNA-seq, none of these methods has yet gained widespread generalizability for detecting and ranking periodically expressed genes. A satisfying understanding of temporal dynamics in mathematically modeling the events of the cell-cycle is needed to generate oscillations. Some mathematical modeling approaches for clock genes, such as cell-cycle or circadian rhythmic data, have been discussed in detail (Ferrell et al., 2011): simple Boolean modeling in a single species-based on-and-off to represent discreteness in regulation from a protein circuit, ordinary differential equations (ODE), delay differential equations, Fourier analysis, and stochastic and partial differential approaches applied in the eukaryotic cell-cycle.

However, the majority of applications have focused on ODE approaches. It has been shown from efforts at modeling the identification of clockwise genes in a periodic time course of microarrays that many computational methods currently cannot be directly applied to RNA-seq data due to the quantification and type of data (i.e., continuous fluorescent intensity vs. discreteness of mapped read counts and gene expression-level quantity vs. gene/isoform-level quantity). Therefore, efficient methods should be well established to analyze periodically temporal dynamics by capturing linear or non-linear dynamic behaviors of modules and interactions of genes during different stages or successive time points in system biology. The identified biological disruption or abnormal pattern on clockwise genes in a timing system is being further investigated as impairments on metabolic regulation (Ferrell et al., 2011; Mazzoccoli et al., 2012). In RNA-seq, researchers in this field strive to develop edge methods that accurately quantify the rhythmical periodic behaviors of genes from large numbers of variables and very few observation structures.

### CO-EXPRESSION AND DIFFERENTIAL EXPRESSION ANALYSIS OVER TIME IN MICROARRAY

Some papers suggested more improved methods to extract core co-regulated patterns of interest from an entire gene set, including some noisy set (Tseng and Wong, 2005; Yuan et al., 2008). Furthermore, cluster analysis may fail to detect changes over a time-series in genes that belong to clusters in which most genes do not change (Bar-Joseph et al., 2001, 2003). There is the perennial question of how many clusters to use as a parameter when applying clustering methods other than the hierarchical clustering approach. Although some groups have been investigating how to choose a proper number of clusters, it is still in question, and arbitrarily varying choices are usually used as initial trials. More specifically, some researchers have proposed gene clustering algorithms for time-series expression in combinations of genes and samples simultaneously by considering the correlated relationship of samples (Luan and Li, 2003; Song et al., 2007). Ernst and Bar-Joseph (2006) also developed the short time-series expression miner (STEM); however, this method is applicable when there is only one biological condition in the time course experiment. Thus, some of the literature on gene clustering methods have highlighted the importance of the identification of co-expressed patterns at the static or dynamic by itself and in a combinatorial way with the detection of differentially expressed individual genes or biological functional pathways, although clustering methods have inherent issues, as discussed above. This section, first of all, focuses on an in-depth discussion of the technical and methodological aspects of the identification of a TDE in time-series classical hybridization technologies. A complete review of other downstream analyses for microarray is beyond the scope of this study and can be found elsewhere (Butte, 2002; Filkov et al., 2002; Ahdesmaki et al., 2005, 2007; Androulakis et al., 2007; Oh et al., 2011; Swan et al., 2011; Nascimento et al., 2012). Due to unavoidable sources of uncertainty from the addition of noise and artifacts on signals, prior to the main downstream analyses, normalizing, filtering, cleaning, and removal of noise and artifacts are crucial initial procedures to produce reliable DEX and results. The overall framework for preprocessing raw microarray data is described in Bolstad et al. (2004), Zahurak et al. (2007), Owzar et al. (2008) and Shakya et al. (2010) by a series of log-data transformations and data quality, normalization to adjust for effects which arise from variations in the microarray before analyses are performed, and treatments of missing values by imputation methods. Some characteristic features of replicated microarray time course data are typically short series, and one classifies a shorter time-series as *k* = 4–10 time points and a longer time-series as 11–20 time points, very often irregularly time-spaced. Compared to RNA-seq, although relatively a microarray platform has more samples, occasionally a few replications with *n* ≤ 5 has been a common standard. Gene expression values at different time points may be inherently correlated, especially if a common reference design is used or a common pool of cells is sampled (Tai and Speed, 2009).

Similar to RNA-seq, in a time course microarray experiment, three main types of structures have been studied so far. The first type is a single-series time course that may have no known particular patterns, as in developmental time courses (Chu et al., 1998; Wen et al., 1998; Tamayo et al., 1999). As in factorial time course design, the second type is a study with different biological conditions at each time point over a time-series, as a plant’s response to a pathogen and the transcriptional response to oxidative stress in the heart and how it changes with age to investigate TDE genes in (Kal et al., 1999; Edwards et al., 2003). The main objective of such previous studies is to identify genes whose temporal expression patterns following response differ between different biological conditions of more than two. The third type is a periodic experiment with cell-cycle and circadian rhythmic data (Cho et al., 1998; Spellman et al., 1998; Storch et al., 2002). As pooling approaches with statistics, LIMMA and ANOVA-based methods have been proposed to address a multi-series time course (Smyth, 2004; Meyer et al., 2012). But as already discussed, such pooling samples do not consider time-dependent structure explicitly; identical results would be obtained even if columns were reordered and there existed obstacles to hinder the power of detection. Other efforts at temporal analysis have been done in regression-based methods by Xu et al. (2002) and Conesa et al. (2006), and the robust Wald statistics by Guo et al. (2003). GeneTxWarper (Criel and Tsiporkova, 2006) is a tool of alignment to analyze and mine gene expression time-series based on dynamic time-warping techniques. The EdgeR R package is a spline-based approach for significance analysis in a time course microarray experiment within one or between several conditions proposed by Storey et al. (2005) and Leek et al. (2006). There is a multivariate approach proposed by Tai and Speed (2006, 2009) and Nueda et al. (2007, 2010), an HMM taking into account time-dependent property by Yuan and Kendziorski (2006) and Yuan et al. (2008, 2011), and an SEA web tool to run multifactorial time-series data developed by Nueda et al. (2007), 2010. Smooth spline methods were used to identify temporally differentially expressed gene patterns in (Song et al., 2007) and (Luan and Li, 2003, 2004; Billups et al., 2009). More recently, Yuan et al. (2011) studied a dynamic time-warping algorithm (DTW-S) to analyze primate brain expression time-series data using a smooth spline function. They compared three different species using a published microarray dataset and showed shift degrees in the human species compared to other species during the development of the prefrontal cortex.

## DEX ANALYSIS IN DIGITAL SAGE, CAGE, AND MPSS EXPERIMENTS

The analysis of gene expression profiles within an organism has been one the most general approaches in molecular biological research to simultaneously monitor large-scale genes and samples in evolution across time points of developmental phenomena or human disease and its treatment over a period of time. As the means for profiling gene expression, following microarray, initial direct sequencing of cDNA libraries, MPSS (massively parallel signature sequencing), SAGE, and CAGE (cap analysis of gene expression) have been popular tools. The main difference to microarrays is that they provide the tag-based expression level quantification and digital measurement technologies to enable the quantification of the expression levels of novel genes and alternatively spliced transcripts without prior knowledge. cDNA microarray has been successfully applied in transcriptome studies, but it provides partial information about abundance based on fluorescent intensity, whereas the expression level in SAGE is quantified by a short sequence-tagging enzyme that gives rise to 15-bp tags to uniquely identify a transcript (Velculescu et al., 1995; Brenner et al., 2000; Pollock and Grime, 2002). Similarly, MPSS technology has the advantage that does not require prior information of the sequences of the transcripts expressed in the cell or tissue to be compared. And the deepCAGE, with short 20-nucleotide sequence tags, has been used uniquely to identify the promoter of each transcript and its expression measurement sites on a genome-wide scale. In contrast, MPSS and SAGE produce tag counts or fragments as locating at the 3′ end of the transcript and do not identify the promoter that might be mapped more upstream in the genome sequence (Yeh et al., 2002). As variants of SAGE, 5′ SAGE using oligo-capping to generate tags and superSAGE allowing isolation of 26-bp tag fragments from expressed transcripts (Matsumura et al., 2011) have also been applied. But they are not currently used as solutions in gene expression profiling, since they do not have sufficient depth of coverage in libraries, and the quantification of low expression levels of transcripts is not reliable.

Some studies associated with the identification of altered changes of expression in temporal or/and spatial patterns initially investigated using digital expression. But applications on the proper methods to facilitate the temporal analysis of large-volume data generated by digital technologies have been poorly addressed when compared to the comprehensive gene expression microarray approach, which has been the most commonly used technology so far. The next-generation sequencing currently revolutionizing the transcriptome field thus presents advantages and a great potential over the previous technologies by allowing for more in-depth studies. Detailed reviews of methods for identifying DEX in early digital technologies can be found in (Baggerly et al., 2003; Lu et al., 2005; Gilchrist et al., 2007; Zaretzki et al., 2010). Through tag-based approaches, including the SAGE-seq, deepCAGE, and MPSS approaches, direct sequencing of cDNA libraries has been applied in transcriptome studies as an alternative to microarrays by not relying on genome annotation for prior probe selection. Investigators have demonstrated transcriptional profiles in a variety of developmental processes, including plant and animal model development, embryogenesis, defense responses to pathogens, and response to drug treatment. More recently RNA-seq, with its improved technical quality, has replaced methods used to identify developmentally related genes or drivers in tumor progression or significant differences between given experimental conditions. Along with the advent of new technologies to explore gene expression profiling, the use of methods of testing hypotheses by developing a robust and precise approach to fully account for underlying static or even dynamic temporal data structure has been investigated. Transcriptomic profiles are defined by a class of high-throughput approaches, classical microarray, the initial digital gene expression measurement such as SAGE, and more recently RNA-seq, offering considerably greater throughput, which enables the expression-level measurement of the abundance of tens of thousands of transcribed RNAs in a given sample to be analyzed (Okaty et al., 2011).

## INVESTIGATING RNA-seq TIME-SERIES DATA USING DYNAMIC DEX METHODS

The newly emerging RNA-seq technology enables the study of the transcriptional program of various types of time-series experimental designs underlying the development and evolution of complex organisms. A more robust and precise methodology is needed to fully understand the complex temporal patterns that contribute to biological questions in their developmental systems so as to better understand the causal relationship between gene expression and time. This is not a trivial problem and makes it difficult to study them without a sophisticated statistical method. The identification and characterization in RNA-specific time-series expression profiles has been a long-standing challenge. As an alternative, static methods, limited but intuitive, have been applied for RNA-seq time-series data (Kvam et al., 2012; Rapaport et al., 2013; Soneson and Delorenzi, 2013). The current analytical limitation of static methods will be overcome with more rigorous dynamic methods that account for sequential correlation on time-series expression profiles as the cost of sequencing continues to decline and the appropriate number of samples and time points become available.

Accordingly, the issue of development of methodologies in RNA-seq time-series data has emerged as a new syndrome in this field, following by statistical methods to detect changes of expression in pairwise comparisons. Prior to exploration and deep discussion on dynamic methods, an understanding of the pros and cons of different static methods is critical to enumerating the specific benefits of the temporal aspects and the future direction of applications of robust new methods of dynamic time-series RNA-seq data in a given biological context. The advantages and limitations of the existing static methods in the identification of DEX were comprehensively reviewed in the previous sections.

The important question of how many time points (data points) and replicates should be used to identify a particular temporal expression pattern might be raised in the beginning of the experiment to increase power and efficiency, since the cost of sequencing data in time-series is not insignificant (Auer and Doerge, 2010; Fang and Cui, 2011; Pauli et al., 2012; Busby et al., 2013).

Since RNA-seq time-series gene expression profiles in biological systems commonly contain a short series of time points, as extended studies from this current study show, methodologies based on gene-to-gene interactions sharing information across genes will gain more sensitivity in detecting genes whose changes of DEX are not identified marginally in the gene-by-gene univariate dynamic approaches presented in this review. In addition, it is not still clear how to assess time course experimental design with multi-factors (and/or multi-groups) in a small sample setting or large-scale longitudinal data with repeated measurements in clinical applications. For example, ones are interested in identification of temporally differentially expressed genes characterized from different enzyme effects to stimulus-response experiments of human diseases. In the particular time course data with multiple parameters to distinct levels of experimental or biological factors, such as different tissues, strains, or drug treatments in microarray, ANOVA and LIMMA have generally been applied in relatively large experiments compared to RNA-seq. However, to date RNA-seq assays in a multi-series of time course, a factorial time course experiment has not been explored in statistical methodology perspectives, even though such studies play a pivotal role in revealing temporal mechanisms of expression from disease-specific target genes with the stimulus of drug treatments. Thus, RNA-specific methodologies of identifying temporal expression in stimulus-response experimental settings will offer a range of specialized applications that have not been available in conventional approaches to gene therapeutic effects. As an *ad hoc *approach, our group is currently developing a statistical method to consider factorial time course experiments (multi-series time course) as well as identification of periodically regulated genes. Another interesting topic to be addressed in RNA-seq is genetic regulatory networks at alternative splicing (AS) diversity and gene level quantification. More recently, (Stower, 2012) also pointed out the importance of post-transcriptional studies in a time course and evidence for the widespread association of incompletely spliced transcripts. Wood et al. (2007) emphasized RNA-directed therapy strategies, including altered processing of the target pre-mRNA transcript and the reprogramming of genetic defects through mRNA repair, comparing it to conventional gene therapies as an emerging field in clinical applications of genomics.

Characterization of temporal dynamics at AS diversity will soon be a very promising and prominent research area by taking a look at transcript variants individually other than unified gene level quantification. In addition, in order to study cell-cycle or circadian rhythmic variations with periodicity, an appropriate statistical methodology must be selected to identify significantly cell-cycle-regulated (or periodically expressed) genes of the genome in a given organism. Currently there are no effective methods of defining the subset of predominantly periodically expressed genes in RNA-seq genome-wide analysis from factorial periodical time course experiment with conditions at a time.

## CONCLUSION AND DISCUSSION

From a DEX perspective as steady-static methods, importantly, prior to detection of temporal difference a normalization procedure must be incorporated to adjust various artifacts from experiments. In order to be comparable between distinct intra- and inter-samples, although intra-samples at a static time point are assumed to be independent, the challenge is to incorporate the fact that a gene’s expression at time t is dependent on its expression at the previous time point t-1. This remains elusive because the normalization methods all assume that samples are independently distributed, which is not true in time-series reality. It has become apparent that reliable detection of the DEX between two different or multiple groups at a static stage (or time point) is the key to understanding complex biological functions and identifying known and novel disease-specific genes between distinct groups such as those that lead to various types of tumors. For now, the importance of detection of DEX in dynamic ways to provide practical solutions to comprehensively exploiting temporal RNA-seq data is emphasized by the growing popularity of time-series experimental studies on a system level for characterizing the temporal orchestration of behaviors as a function of a time effect in gene regulation of complex biosystems. Through DEX based on ranking individual genes, numerous candidate genes with disease effects in age or developmental progress can be detected. However, the exact mechanisms underlying the influence of these genes and the relationship between individual genes in temporal regulation must be further examined.

Analysis of time-series RNA-seq data is still at an immature stage in terms of development and application of methods to decipher the complexity of a series of observations in time-series representations. Most studies in RNA-seq time-series data so far have applied methods that were originally developed for static expression profiles without respect to time using simple pairwise comparisons in RNA-seq data or for time-series methods in microarray after variance-stabilizing transformation. The identification and analysis of static gene expression profiles in RNA-seq have become routine, and the rapid growth of time-series studies for developmental biology and disease processes in clinical applications imposes issues for traditional methods of analyzing dynamic system mechanisms. In general, the limited number of time points and replications in experimental design are due to the expense of sequencing and the limited number of available biological RNA-samples. This difficulty in obtaining the proper number of samples for the many time points and biological replicates prevents investigators of statistical methods from establishing standards for time-series analyses due to large-scale dimensionality with the small number of observations. And the corresponding large number of parameters causes a singularity of matrix, over-fitting, and misleading results with false discovery rates. To overcome this, short-time-series data can be dealt with by borrowing meaningful information across genes in shrinkage methods, incorporating prior information for representative temporal patterns or sequential correlation of gene expression between consecutive time points in Bayesian approaches, incorporating prior information with other genomic levels with Chip-seq and methylation data, and collecting meta time-series data, although the challenges regarding the heterogeneity of data remain.

The advanced, powerful statistical methods used to identify temporal expression in a variety of RNA-seq time course data will be useful both for web-lab biologists/clinicians and computational/statistical analysts who want to understand altered gene expression over time, for gaining insights into biological systems and for obtaining rich information from the data. Analyses of temporal gene expression patterns may soon result in improvement in the diagnosis and treatment of tumor progression or neurodegenerative brain diseases and other human diseases with a time window and experimental treatment conditions in gene expression profiling. This comprehensive discussion provides the first systematic review of methodologies on the identification of dynamic expression profiles in time-series RNA-seq and forms the foundation for future genetic, genomic, and developmental evolutionary studies related to human disease and health. Understanding dynamic transcriptomes is crucial to understanding the mechanisms of cell differentiation and ultimately providing therapeutic immune system solutions or for characterizing cell signaling and mitochondrial dynamics in neural degenerative diseases such as Alzheimer’s and Parkinson’s from gene expression profiles, although RNA-seq has not yet been extensively utilized to characterize these diseases.

The pros and cons of several static methods of identifying differentially expressed genes are listed here, including simple pairwise comparison and multi-group comparison, and the limitations of such methods in discerning temporal and spatial transcript structure and analyzing transcriptome complexity in dynamics are presented. Thus dynamic methodologies, including the periodic time course proposed here, will provide critical insights from simple short time course to retrospective studies of disease patients according to clinical characteristics. The approaches can be applied in disease-related time course RNA-seq transcriptome data or other count featured -omics data, such as the initiation and progression of the immune response in dynamics behavior in a given patient and the complexity and dynamics of the human brain. In microarray, a longitudinal breast cancer study identifying the gene expression profiles compared between enriched cell populations and whole bone marrow from normal volunteers and breast cancer patients after neoadjuvant chemotherapy treatment could be an ideal example for defining the correlation of the disease status, response to treatment and survival in course of the study (Watson et al., 2007). This study used enriched cells from bone marrow samples of breast cancer patients before treatment or at 1-year follow-up and analyzed by integrating total of 87 data sets. The expression of transcripts specifically detected in enriched cell populations from breast cancer patients was correlated with 1-year clinical outcome using quantitative reverse transcription-PCR in an independent cohort of bone marrow samples.

Through currently the most attractive technology, RNA-seq, identification of temporal changes of gene expression will provide a potential avenue for future studies of genetics, genomics, system biology in developmental process and time-dependent observed data such as aging, parkinson’s or Alzheimer’s disease and for screening patients with tumor progression and infectious dynamic disease using the new technology of RNA-seq time course experiments in the various genomes.

More specifically, techniques are described here for identifying temporal patterns that take into account the autocorrelation and Markovian assumption that a time-series random variable typically exhibits high level of sequential correlation and the current expression levels are dependent on past expression profiles. The more informative dynamic methodologies considering correlation structure across time points to identify temporal patterns will be widely used with the advantages in RNA-seq ahead.

To overcome a general lack of dependent assumptions on existing methods and owing to small size of observations in RNA-seq settings, statistically rigorous and validated approaches for time course will lead to find dynamic response markers on gene expression profiles by accounting for proper assumptions, robustness, and biological interpretations in gene functional pathways of system biology at the most perturbed time point. Moving on temporal dynamic analysis, the review describes the first systematic and comprehensive identification methods of static and temporal dynamic patterns in RNA-seq transcriptomic data, including array-based experiments, the very beginning of sequencing-based experiments.

## CLOSING REMARKS

The comprehensive enterprise of DEX analysis in a class of high-throughput technologies was discussed by highlighting the identification of DEX in static and temporal dynamics in RNA-seq time-series, which have not been explored in statistical modeling approaches to identify temporal expression, implying quantitative biological scenarios in the biomedical research community. To make this connection, this review is intended to be a guide to the choice and use of a suitable method in a given study and to lead to a significant paradigm shift in RNA-seq methodology. It can further advance our understanding of hypotheses in directing a mathematical relationship of system biological behaviors and statistical modeling because all static existing methods are restricted to direct applications for time-series dynamic data without any adaptations of modeling and estimation. Without appropriate statistical measures in temporal analysis, almost any static approach will yield significant genes, including a large number of false discoveries. Furthermore, by incorporating the advantages of RNA-seq, in a parallel manner, AS events and allelic specific expression will be also of value in addressing the consequences of mis-regulation of splicing and allele in spatial and temporal time-series expression profiling in the context of human disease development. As an important extension remark of these issues, a mathematical graphical model is being developed in biological systems over time on how to infer the temporal dynamics of underlying networks in RNA-seq time-series data. For example, neuronal migration during development of the cerebral cortex requires particular exon-skipping events in a given transcript, and identification of the specific transcripts which undergo a developmentally induced AS switch in migrating neurons will play a critical role in defining the dynamics of expression profiles and the intimate connections between the regulation of AS and development (Garcia-Blanco et al., 2004; Wang, 2007; Wang et al., 2008; Douglas et al., 2009; Douglas and Wood, 2011; Grabowski, 2011). Many computational and statistical tools are currently in development, and many issues must be addressed in RNA-seq, including quantification of expression levels, normalization to adjust for experimental biases and technical variability between replicates, DEX between groups, comparative and integrative analysis of various methods, etc. One of the main interests of findings in this study is the inference of TDE, and characterization of temporal patterns will play a predominant role in perspectives of statistical methodologies in temporal analysis via RNA-seq. In this manuscript, comprehensive review of statistical methodologies at a static and dynamic were provided as statistical frameworks to define and uncover dynamic temporal profiling for time course RNA-seq experiments as static expression profiles that will be of great value in characterizing the regulation of gene expression during a specific time period.

## AUTHOR CONTRIBUTIONS

Sunghee Oh conceived the reviews and wrote the manuscript, Sunghee Oh, Seongho Song, Nupur Dasgupta, and Gregory Grabowski contributed to critical discussion on mathematical approaches and biological aspects in transcriptome dynamics.

## Conflict of Interest Statement

The authors declare that the research was conducted in the absence of any commercial or financial relationships that could be construed as a potential conflict of interest.
